# Tissue Renin-Angiotensin System (tRAS) Induce Intervertebral Disc Degeneration by Activating Oxidative Stress and Inflammatory Reaction

**DOI:** 10.1155/2021/3225439

**Published:** 2021-08-06

**Authors:** Kaiqiang Sun, Xiaofei Sun, Jingchuan Sun, Yan Jiang, Feng Lin, Fanqi Kong, Fudong Li, Jian Zhu, Le Huan, Bing Zheng, Yuan Wang, Weiguo Zou, Lu Gao, Ximing Xu, Jiangang Shi

**Affiliations:** ^1^Department of Orthopedic Surgery, Changzheng Hospital, Navy Medical University (Second Military Medical University), No. 415 Fengyang Road, Shanghai 200003, China; ^2^Department of Oral and Maxillofacial-Head Neck Oncology, Shanghai Jiao Tong University School of Medicine, Shanghai, China; ^3^State Key Laboratory of Cell Biology, CAS Center for Excellence in Molecular Cell Sciences, Shanghai Institute of Biochemistry and Cell Biology, Chinese Academy of Sciences, University of Chinese Academy of Sciences, Shanghai 200031, China; ^4^Department of Physiology, College of Basic Medical Sciences, Navy Medical University (Second Military Medical University), Shanghai 200433, China

## Abstract

Lumbar intervertebral disc degeneration (IDD) has been the major contributor to low back pain (LBP). IDD is an chronic inflammation process, with the activation of plentiful inflammation-related cytokines and ECM degradation-related enzymes. In the past few years, hypertension has been reported to correlate with LBP. In addition, the local tissue renin-angiotensin system (tRAS) has been identified in multiple tissues, including the spinal cord, skin, kidney, heart, and bone. Recently, tRAS has also been established in both bovine and human intervertebral disc tissues, especially in the degenerated disc tissue. However, the exact of tRAS and IDD remains unknown. In this present study, proteomic analysis, molecular biology analysis, and animal model were all used. Firstly, we revealed that tRAS was excessively activated in the human degenerated intervertebral disc tissue via proteomic analysis and molecular biology analysis. Then, in vitro experiment suggested that Ang II could decrease the cell viability of human NP cells and promote NP cell apoptosis, senescence, oxidative stress, and NLRP3 activation in human NP cells. In addition, Ang II could also trigger degeneration and fibrosis phenotype in human NP cells. Finally, the animal model demonstrated that the local activated ACE/Ang II axis in the NP tissue could accelerate IDD in aging spontaneously hypertensive rats (SHR). Collectively, the degenerated intervertebral disc tissue showed excessively activated tRAS, and local activated tRAS could induce NP cell senescence, apoptosis, oxidative stress, and inflammatory reaction to promote IDD. These biological effects of Ang II on human NP cells may provide novel insight into further treatment of IDD.

## 1. Introduction

Low back pain (LBP) has been the major contributor to hospitalization in patients with degenerative lumbar diseases, which brings enormous social and economic burdens for patients and their families worldwide [1]. Of the massive pathogenic factors, lumbar intervertebral disc degeneration (IDD) has been the mostly studied. Anatomically, the normal intervertebral disc is consist of gel-like inner nucleus pulposus tissue (NP), surrounding annulus fibrosus tissue (AF), and cartilaginous endplates structure (EP), which connects adjacently superior and inferior vertebral bodies [2, 3]. In general, IDD is an age-related biological process. However, accumulating evidence has suggested that multifactorial pathogenesis can initiate or accelerate IDD, such as repetitive mechanic load, infective diseases, trauma, and genetic predisposition. During the process of IDD, various pathological changes occur in the microenvironment of the NP tissue, such as cell apoptosis, cell senescence, inflammatory reaction, and oxidative stress, which will result in increased catabolic reaction and decreased synthetic reaction [4]. These abnormal biological processes will finally cause IDD-related diseases, such as spinal canal stenosis, adjacent segment degeneration (ASD), and spinal instability [4].

Clinical treatments have been well built based on the duration of symptoms, age, comorbidities, severity of IVD, compression of neural elements, and spinal stability. In early stage, patients with symptomatic IDD could be well controlled via medication therapy, such as vitamin B12 combined with nonsteroidal anti-inflammatory drugs [5]. However, surgical interventions are frequently recommended, especially for patients with serious neurological dysfunction [5]. Nevertheless, the long-term surgical effects could be ineffective, and the occurrence of ASD cannot be ignored [6]. In recent years, basic research therapy has attracted substantial interest. Various molecular treatments targeted at inhibiting the inflammatory responses and oxidative stress have been established, including gene therapy, growth factor therapy, and cell therapy [3, 5, 7]. However, due to the limitation of the exact mechanism of IDD and further clinical trials, these therapies above have not yet acquired satisfactory generalization. Thus, more work is still required to explore the potential mechanism of IDD.

Hypertension, as a common chronic disease, has been reported to be a risk factor for low back pain, and antihypertensive medication may attenuate this association [8, 9]. However, the potential mechanism of hypertension involved in IDD remains unknown. The regulatory effects of the renin–angiotensin system (RAS) on vasoconstriction, blood pressure, and electrolyte balance during hypertension have been well established. Ang II, as a key mediator in hypertension, is mainly produced from angiotensinogen (ATG) via angiotensin-converting enzyme (ACE) [10]. AT1 receptor (G protein-coupled receptor) is the main biological mediator of Ang II, and the binding of Ang II to AT1 will enhance the generation of ROS, the release of pro-inflammatory cytokines, and, the accumulation of M1 macrophages [10, 11]. However, plentiful researches have suggested the circulatory homeostasis-independent biological effects of Ang II in multiple organs or tissues in the past decades. Li et al. ever reported that Ang II could induce mitochondrial oxidative stress and DNA damage in osteoblasts to accelerate the process of osteoporosis [12]. Ang II could also enhance the infiltration of inflammatory cells, such as macrophages, into local tissue, and cause tissue damage [13]. Andrew et al. previously demonstrated that macrophage Ang II receptor could trigger chronic neuropathic pain [14]. In addition, Ang II is also involved in degeneration-related diseases, such as osteoarthritis and Alzheimer's disease [10, 15, 16]. Therefore, a new term “Tissue Renin-Angiotensin System (tRAS)” has been proposed. Interestingly, Morimoto et al. firstly found tRAS components in the normal rat intervertebral disc tissue at both mRNA and protein levels [17]. Further, Li et al. confirmed the existence of tRAS components in the human disc tissue and found that within tRAS-positive disc samples, AGT, matrix-metalloproteinases 13/3 (MMP3/13), IL-1, and other inflammatory cytokines were highly expressed [18]. These results above indicated a potentially critical role of tRAS in IDD. However, the correlation between the activation of tRAS and the process of IDD and the exact molecular mechanisms of Ang II contributing to IDD have not yet been fully elucidated.

This present study is aimed at revealing (1) the correlation of activated tRAS and the degree of IDD in the human disc tissue, (2) the potential biological effects of Ang II on human NP cells and related pathological mechanisms in vitro, and (3) the long-term effects of local activated tRAS in the NP tissue on the process of IDD in vivo. The results will provide new information for future investigation of hypertension and IDD.

## 2. Methods and Materials

### 2.1. Acquisition of Patients' Samples

This study was approved by the ethic board of our institution, and the written informed consent was signed by all the participants enrolled in this study. Lumbar intervertebral disc tissue was acquired intraoperatively from twenty-two patients (mean age: 56 ± 12 years; range: 23-78 years) who accepted surgical fusion due to spine-related diseases (trauma and intervertebral disc herniation) in our institution. According to preoperative Pfirrmann grades on MRI, the disc tissue was divided into the nondegenerated group (grade II), moderately degenerated group (grade III), and severely degenerated group (grade IV). Disc tissue from ten patients including grade II (five cases) and grade IV (five cases) was used to perform TMT quantitative proteomic analysis. In addition, disc tissue from three patients (grade II) was used to isolate NP cells in vitro.

### 2.2. Experimental Protocols

Male WKY rats and SHR with age of 8 weeks (weighed 250-350 g) were obtained from Vital River Laboratory Animal Technology and kept in animal room with 12-h cycle of light and dark. All experiments were approved by the Animal Care and Use Committee of Navy Medical University.

### 2.3. Proteomic Analysis

The intervertebral disc tissue (nucleus pulposus as much as possible) was collected from ten patients (five cases with grade II and five cases with grade IV). The details of proteomic analysis have been described previously, with the experimental workflow in supplementary file (supplementary Figure 1) [19, 20]. Briefly, after collection of the disc sample, the proteins were extracted, alkylated, and digested. After SDS-PAGE separation, the proteins underwent tandem mass tag (TMT) labeling based on the instructions (Thermo Fisher Scientific, Waltham, MA, USA). Subsequently, the TMT-labeled samples underwent high pH reversed-phase peptide fractionation using kit (Thermo Fisher Scientific, Waltham, MA, USA), followed by LC-MS-MS data acquisition and bioinformatic analysis (GO and KEGG).

### 2.4. Acquisition and Culture of Human NP Cells

This method has been reported in our recent study [2]. Briefly, the collected intervertebral disc tissue was transported to laboratory using 0.9% sterile saline within two hours. Then, the tissue was washed 3 times using PBS (Servicebio, Wuhan, China) in super clean bench. Next, gel-like NP tissue was separated and digested using 0.25% Trypsin-EDTA (C0209-100 mL, Beyotime, Shanghai, China) for 30 minutes and 0.2% collagenase type II (Invitrogen, USA) for another 1 h at 37°C under a shaker (70 r/min). Finally, the isolated NP cells were resuspended in complete culture medium (Gibco; Thermo Fisher Scientific, Inc.) and cultured in a 37°C incubator. At the third, half of the culture medium will be replaced by complete medium. Five days after isolation, the spindle-shaped NP cells will move out and adhered at the bottom which we called passage 0. When the cell density reaches about 80%, the NP cells could be used further experiments.

### 2.5. Cell Viability Assay

After seeding in a 96-well plate (4 × 10^3^ cells/well) for 24 hours, cells were incubated with Ang II (10^−4^-10^−10^ M) for another 24 hours. On the third day, 110 *μ*l CCK 8 detection solution (10 *μ*l CCK8 solution and 100 *μ*l DF-12 medium) was added into every single well and coincubated with NP cells for another 1 hour at 37°C. The absorbance of every well was measured with a wavelength of 450 nm on an absorbance microplate reader (Bio-Tek, USA).

### 2.6. Real-Time Quantitative PCR (Polymerase Chain Reaction)

This method has been described in previous study [2]. After acquisition of mRNA from NP cells, total mRNA (100 ng/*μ*L) was reversed using HiScript ® III RT SuperMix for qPCR Kit (R323-01, Vazyme, Nanjing, China). Then, the acquired cDNA was quantified using SYBR qPCR Master Mix (Q711-02, Vazyme, Nanjing, China) on a ABI 7500 Real-Time PCR system (Applied Biosystems, Foster City, USA). GAPDH was used as the normalization, and all reactions were run for three times. Relative mRNA amount was quantified using the formula: 2^−△△Ct^. The primer sequences used are presented in Table 1.

### 2.7. Immunohistochemical Analysis

This method has been described previously [2]. Intervertebral disc samples were sectioned, embedded with paraffin, and deparaffinized, followed by blocking the endogenous peroxide using 3% hydrogen peroxide. Then, the sections were incubated in 5% blocking solution for 60 min and incubated with primary antibody against ACE (24743-1-AP, 1 : 500, Proteintech, China) overnight at the temperature of 4°C. Subsequently, HRP-conjugated secondary (GB23303, Servicebio, Wuhan, China) was used to binding primary antibody, followed by hematoxylin stanning. Images were captured using light microscopy (Olympus, Japan).

### 2.8. Immunofluorescence Analysis

Our previous study also described this method in detailly [2]. After intervertebral disc sections or NP cells were well prepared, 0.1% Triton X-100 was employed to permeate samples for 5 min. Then, samples were blocked with 3-5% BSA for 60 min at 37°C. Then, the samples were incubated using primary antibody against *γ*H2AX (GB111841, 1 : 200, Servicebio, Wuhan, China), aggrecan (GB11373, 1 : 500, Servicebio, Wuhan, China), collagen type II (GB14073, 1 : 500, Servicebio, Wuhan, China), iNOS (GB11119, 1 : 1000, Servicebio, Wuhan, China), collagen type I (GB11022, 1 : 500, Servicebio, Wuhan, China), NLRP3 (GB11300, 1 : 1000, Servicebio, Wuhan, China), CD206 (#24595, 1 : 800, Cell Signaling Technology, Inc. USA), MMP3 (GB11131, 1 : 500, Servicebio, Wuhan, China), AGT (ab108334, 1 : 200, Abcam, USA), Nrf2 (340675, 1 : 500, Zenbio, Chengdu, China), and p65 (GB11142, 1 : 200, Servicebio, Wuhan, China) at 4°C overnight. At the second day, samples were treated with fluorescence secondary antibody (GB22303, GB21301, Servicebio, Wuhan, China) for 1 h in the dark room. The nuclei were staining with DAPI solution (G1012-100ML, Servicebio, Wuhan, China). The fluorescence was detected using fluorescence microscope (Olympus, Japan).

### 2.9. Terminal Deoxynucleotidyl Transferase dUTP Nick-End Labeling (TUNEL) Assay

Our previous study also described this method detailly [2]. After intervertebral disc sections or NP cells were well prepared, 0.3% Triton X-100 was applied to permeating samples for 10 min. Then, the TUNEL detection solution was prepared with 5 *μ*l TDT enzyme and 45 *μ*l fluorescent labeling solution for each sample. After washing the sample twice using HBSS/PBS, the TUNEL detection solution was incubated with samples in a 37°C incubator for 60 min. Subsequently, the samples were again washed using HBSS/PBS for 3 times, followed by DAPI solution. Finally, TUNEL-positive cells were detected using fluorescence microscope (570 nm, Olympus, Japan).

### 2.10. Detection of Reactive Oxygen Species

Our previous study also described this method detailly [2]. Human NP cells were washed using PBS for 3 times, followed by staining with none-FBS cultural medium solved with 2,7-DHD (S0033S, Beyotime, Shanghai, China) for 20 mins. Then, the NP cells were again washed for 3 times in order to wash out remaining DCFH-DA. The level of ROS would be identified by fluorescence microscope (525 nm, Olympus, Japan).

### 2.11. Safranin O-Fast Green Stanning

The rat caudal disc was fixed using 4% PFA for one day and decalcified using decalcifying fluid for 15-20 days. Then, the samples were embedded using paraffin. After dehydration, the samples were incubated with Safranin O Fast Green (G1053, Servicebio, Wuhan, China). Typical images were randomly captured using light microscopy (Olympus, Japan).

### 2.12. Western Blot

Our recent study has described this method detailly [2]. The primary antibodies used in this present study included NOX2 (ab129068, 1 : 2000, Abcam, USA), AT1 (381666, 1 : 1000, Zenbio, Chengdu, China), ACE (24743-1-AP, 1 : 1000, proteintech, China), Bax (200958, 1 : 1000, Zenbio, Chengdu, China), MMP13 ((820098, 1 : 1000, Zenbio, Chengdu, China), C-caspase-3 (#9664, 1 : 1000, Cell Signaling Technology, Inc. USA), p53 (sc-393031, 1 : 500, Santa Cruz, Bio. Lnc, USA), Klotho (382164, 1 : 1000, Zenbio, Chengdu, China), Bcl2 (381702,, 1 : 1000, Zenbio, Chengdu, China), COX-2 (#12282, 1 : 1000, Cell Signaling Technology, Inc. USA), MMP-3 (380816, 1 : 1000, Zenbio, Chengdu, China), aggrecan (ab36861, 1 *μ*g/mL, Abcam, USA), iNOS (#20609, 1 : 1000, Cell Signaling Technology, Inc. USA), Nrf2 (221102, 1 : 1000, Zenbio, Chengdu, China), HO-1 (#43966, 1 : 1000, Cell Signaling Technology, Inc. USA), type II collagen (collagen II (1 : 1000, ab34712, Abcam), SOD1 (#37385, 1 : 1000, Cell Signaling Technology, Inc. USA), NLRP3 (381207, 1 : 1000, Zenbio, Chengdu, China), ASC (340097, 1 : 1000, Zenbio, Chengdu, China), and GAPDH (5174, 1 : 1000, Cell Signaling Technology, Inc. USA). The secondary antibodies were purchased from Zenbio (380172, 511103, Zenbio, Chengdu, China).

### 2.13. Enzyme-Linked Immunosorbent Assay

The secretory amount of Il-18, IL-1*β*, and NO in supernatant of cultured human NP cells was detected using ELISA kit (ab215539, ab214025, Abcam, USA, and SD0621, Westang, China).

### 2.14. Assay of Mitochondrial Membrane Potential (MMP)

Our previous study also described this method detailly [2]. After human NP cells were treated with Ang II, the prepared JC-1 staining fluid (Beyotime Biotechnology, Inc., Shanghai, China) was added into cells (1 mL/well) and incubated at 37°C for 20 min. After then, the NP cells were washed with buffer solution. Fluorescence microscope (Olympus, Japan) was used to analyze the MMP of NP cells.

### 2.15. Assay of Senescence-Associated *β*-Galactosidase (SA-*β*-gal) Activity

This method has been reported previously [21]. Firstly, SA-*β*-gal staining fixative was added to the treated cells and coincubated for 15 min. After washed by PBS, SA-*β*-gal work solution was added to cells drop by drop and incubated at 37°C overnight, covered by plastic wrap. The SA-*β*-gal staining image was acquired under a light microscope (Olympus, Japan).

## 3. Results

### 3.1. Activation of the Tissue-Renin-Angiotensin-System (tRAS) in the Human Degenerated Intervertebral Disc Tissue

**Figure 1:**
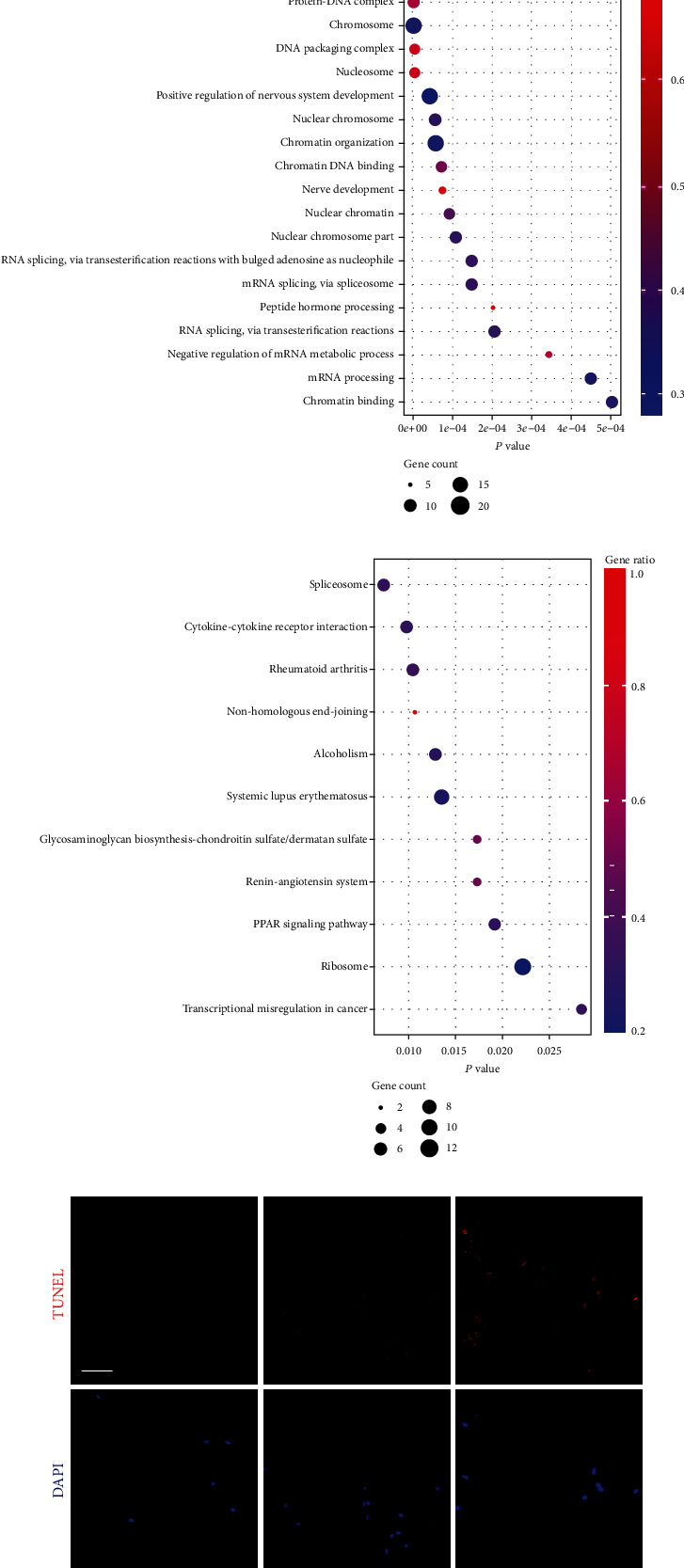
Activation of the tissue renin-angiotensin system (tRAS) in the human degenerated intervertebral disc tissue. (a) The volcano plot of the gene relative expression of NP tissue in the none-degenerated group compared to the severely degenerated group. Pink circles represented upexpressed proteins (*p* < 0.05), whereas black circles suggested proteins without expression differences between these two groups (*n* = 5). (b) The clustering analysis heat map. (c) Top 20 enriched GO terms. (d) Top ten enriched KEGG pathways. (e) TUNEL assay of the human NP tissue among grades II, III, and IV (*n* = 3). (f) Quantitative results of TUNEL-positive cells. (g) Immunohistochemical analysis of the expression of ACE in the human NP tissue among grades II, III, and IV (*n* = 3). (h) Quantitative results of ACE-positive cells. (i, j) Western blot results of the protein expression of tRAS components (ACE and AT1) and MMP13 in the human NP tissue (*n* = 3). Scale bar = 50 *μ*m. ^∗^*p* < 0.05, ^∗∗^*p* < 0.01, ^∗∗∗^*p* < 0.001, ^∗∗∗∗^*p* < 0.0001.

This project was carried out using TMT Quantitative TMT-labeling LC-MS-MS, and totally, 1841 proteins were identified. With 1.2 less or more fold changes (*p* < 0.05) being screened by the criteria, there were 112 upregulated proteins and 79 downregulated proteins in none-degenerated group compared to the severely degenerated group (Figure 1(a)). The altered protein expression profile in these two groups was presented using hierarchical clustering (Figure 1(b)). The Kyoto Encyclopedia of Genes and Genome (KEGG) pathway enrichment analysis revealed the altered proteins with their important signal pathway, including renin-angiotensin system, glycosaminoglycan biosynthesis pathway, PPAR signaling pathway, alcoholism, and systemic lupus erythematosu (Figures 1(c) and 1(d)). Based on gene counts and gene ratio, we focused on RAS. In addition, AGT (P01019), the origin of Ang II, was found to be expressed higher in severely degenerated group, indicating increased activation of tRAS that may correlate with IDD.

We further confirmed the expression changes of tRAS components in the human NP tissue at the molecular level. Based on Pfirrmann grades on T2-weighed MRI, the human intervertebral disc tissue was divided into three groups: grade II, grade III, and grade IV, respectively (*n* = 3). As shown in Figure 1, the ratio of TUNEL-positive nucleus pulposus increased with Pfirrmann grades (Figures 1(e) and 1(f)). In addition, the expression of ACE was also elevated with the increased degree of degeneration of disc (Figures 1(g) and 1(h)). Further, western blot analysis of the NP tissue of disc confirmed the increased tRAS components (AT1 and ACE) in degenerated disc (Figures 1(i) and 1(j)). These results above suggested the activation of tRAS in the human degenerated intervertebral disc tissue.

### 3.2. Inflammatory Cytokines Could Promote the Activation of RAS in Human Nucleus Pulposus Cells In Vitro

**Figure 2:**
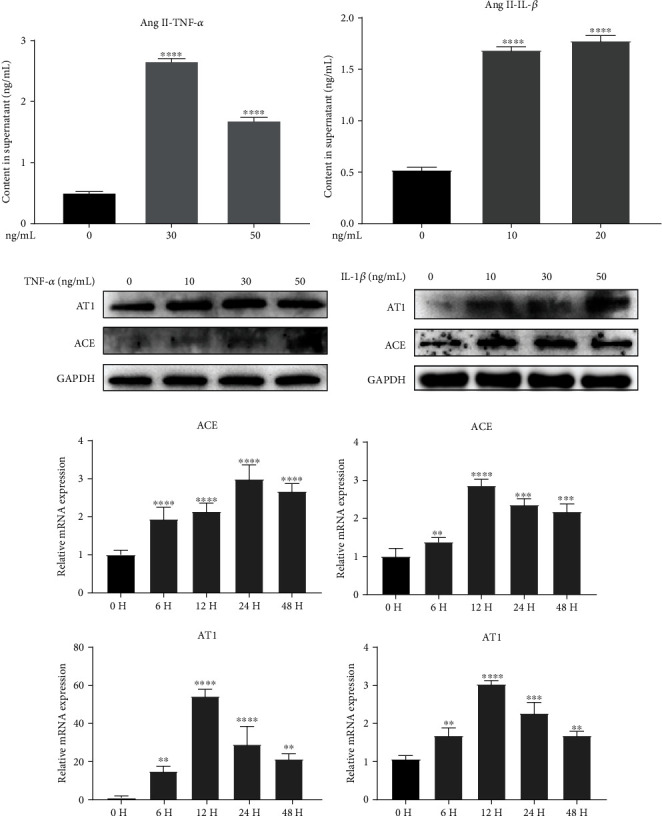
Inflammatory cytokines promoted the activation of tRAS in human nucleus pulposus cells in vitro. (a, b) Secretion amount of Ang II in supernatant of human NP cells induced by IL-1*β* or TNF *α* was quantified by the ELISA assay (*n* = 5). (c, d) Western blot revealed the relative protein expression of tRAS components (ACE and AT1) in human NP cells induced by IL-1*β* or TNF *α* (*n* = 3). (e, f) RT-qPCR quantified the relative mRNA expression tRAS components (ACE and AT1) in human NP cells induced by IL-1*β* or TNF *α* in a time-change manner (*n* = 3). ^∗^*p* < 0.05, ^∗∗^*p* < 0.01, ^∗∗∗^*p* < 0.001, ^∗∗∗∗^*p* < 0.0001.

To further investigate the expression changes of tRAS components in nucleus pulposus, we stimulated human nucleus pulposus with inflammatory cytokines (TNF *α* and IL-1*β*) in vitro, respectively. Firstly, human nucleus pulposus cells treated by either TNF *α* or IL-1*β* secreted higher level of Ang II in supernatant (Figures 2(a) and 2(b) both *p* < 0.05). In addition, the results of western blot suggested that inflammatory cytokines could dose-dependently activate the expression of AT1 and ACE in NP cells (Figures 2(c) and 2(d)). RT-qPCR analysis also showed that inflammatory cytokines promoted the activation of tRAS in nucleus pulposus in a time-dependent manner, especially at 12-24 h following inflammatory stimulation (Figures 2(e) and 2(f)).

### 3.3. Angiotensin II Triggered Human NP Cell Senescence in a Dose-Dependent Manner

**Figure 3:**
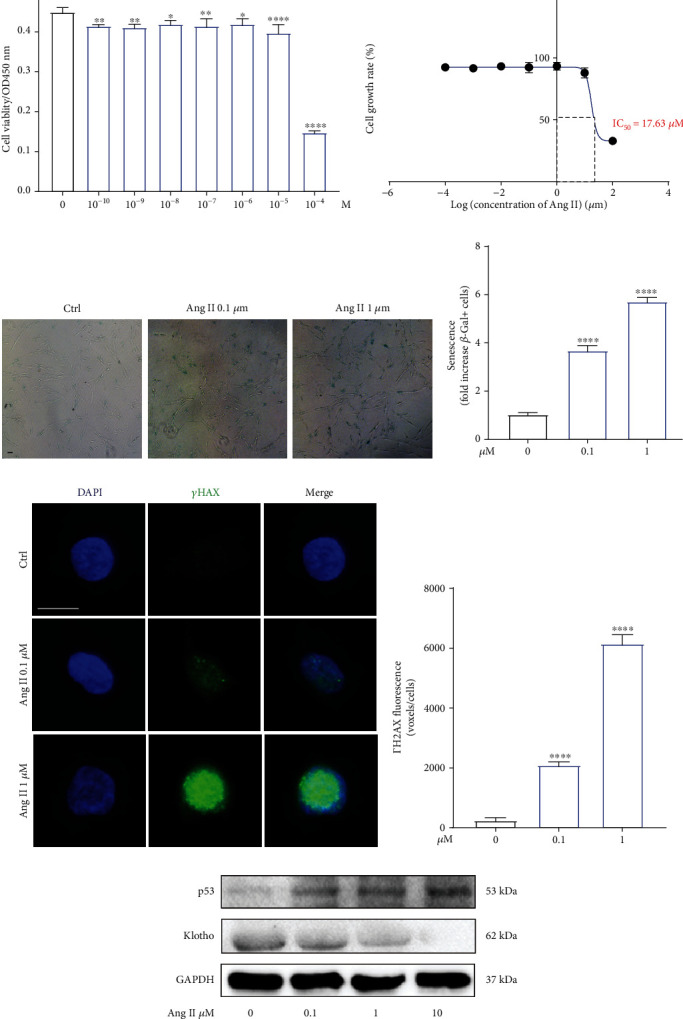
Angiotensin II triggered human NP cells senescence in a dose-dependent manner. (a) The SA-*β*-gal staining of human NP cells induced by Ang II (*n* = 3). (b) Quantitative results of SA-*β*-gal-positive NP cells in different groups (*n* = 3). (c) Immunofluorescence results for the expression of *γ*H2AX in human NP cells treated by Ang II (*n* = 3). Scale bar = 20 *μ*m. (d) The expression of *γ*H2AX was quantified as averaged fluorescent voxels/cell (*n* = 3). (e) The protein expression of senescence-related markers, p53, and Klotho in human NP cells induced by Ang II (*n* = 3). *p* < 0.05, ^∗∗^*p* < 0.01, ^∗∗∗^*p* < 0.001, ^∗∗∗∗^*p* < 0.0001.

As shown in Figure 3, Ang II could decrease the cell viability of NP cells in a dose-dependent manner, with the IC_50_ being 17.63 *μ*M (Figures 3(a) and 3(b)). SA-*β*-gal has been proposed as a marker of cell senescence [22]. After treated by Ang II (0, 0.1, and 1 *μ*M) for 24 h, the staining for SA-*β*-gal in human nucleus pulposus was significantly enhanced with increased concentrations (Figures 3(c) and 3(d)). In addition, before the occurrence of cell senescence, DNA damage will be firstly triggered, which has considered as presenescence responses to stress. Excessive accumulation of phosphorylated histone H2AX (*γ*H2AX) at the injured sites is the typical characteristic of DNA damage [23]. The immunofluorescence analysis demonstrated that Ang II increased the amount of *γ*H2AX distribution at nuclear DNA (Figures 3(e), 3(f), and 3(d)). Consistent with the results above, Ang II also increased the expression of proaging protein, p53, and decreased the expression of antiaging protein, Klotho (Figure 3(g)). Taken together, Ang II could promote human NP cells senescence in vitro.

### 3.4. Angiotensin II Decreased Cell Viability and Induced Apoptosis in Human NP Cells

**Figure 4:**
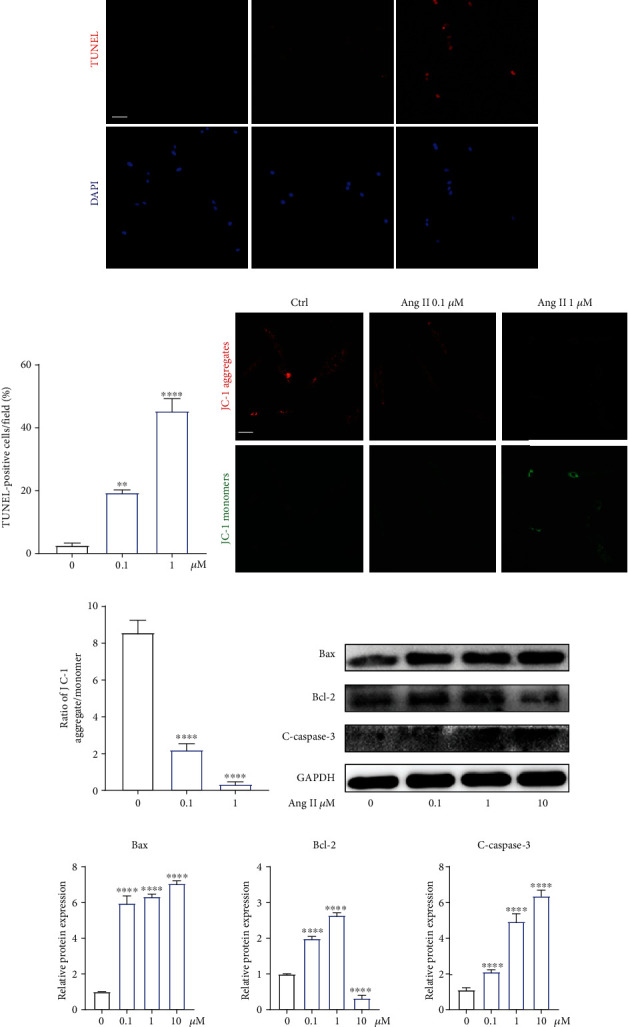
Angiotensin II decreased cell viability and induced apoptosis in human NP cells. (a) The effects of Ang II with different concentrations on human NP cells (*n* = 5). (b) The IC_50_ curve of Ang II for human NP cells. (c) Apoptotic human NP cells were presented using the TUNEL assay (*n* = 3). Scale bar = 50 *μ*m. (d) TUNEL-positive cells were quantified (*n* = 3). (e) The effect of Ang II on the mitochondrial membrane potential of NP cells was detected by JC-1 staining (*n* = 3). (f) The ratio of JC-1 aggregates (red) to monomers (green) was quantified (*n* = 3). Scale bar = 50 *μ*m. (g) The expression of apoptosis-related proteins was analyzed by western blot (*n* = 3). (h) The expression of apoptosis-related proteins was quantified (*n* = 3). *p* < 0.05, ^∗∗^*p* < 0.01, ^∗∗∗^*p* < 0.001, ^∗∗∗∗^*p* < 0.0001.

To further examine the biological effects of Ang II on human NP cells, we evaluated the change of NP cell viability after being exposed to Ang II. The TUNEL assay suggested the proapoptotic effect of Ang II on NP cells (Figures 4(a) and 4(b)). Mitochondrion plays a critical role in regulating cell apoptosis [24]. Therefore, we further evaluate the changes of mitochondrial membrane potential (MMP) and found that Ang II could also decrease MMP in a dose-dependent manner (Figures 4(c) and 4(d)). Proapoptosis markers, cleaved-caspase 3 and Bax, and antiapoptosis marker, Bcl-2, have been the vital components that are involve in the mitochondria-related pathway [25]. Our western blot results revealed that Ang II increased the expression of cleaved-caspase 3 and Bax and suppressed the expression of Bcl-2 (Figures 4(e) and 4(f)). The results above confirmed the suppressive effect of Ang II on cell viability and the promotive effects of Ang II on cell apoptosis.

### 3.5. Angiotensin II Induced Degeneration and Fibrosis Phenotype in Human NP Cells In Vitro

**Figure 5:**
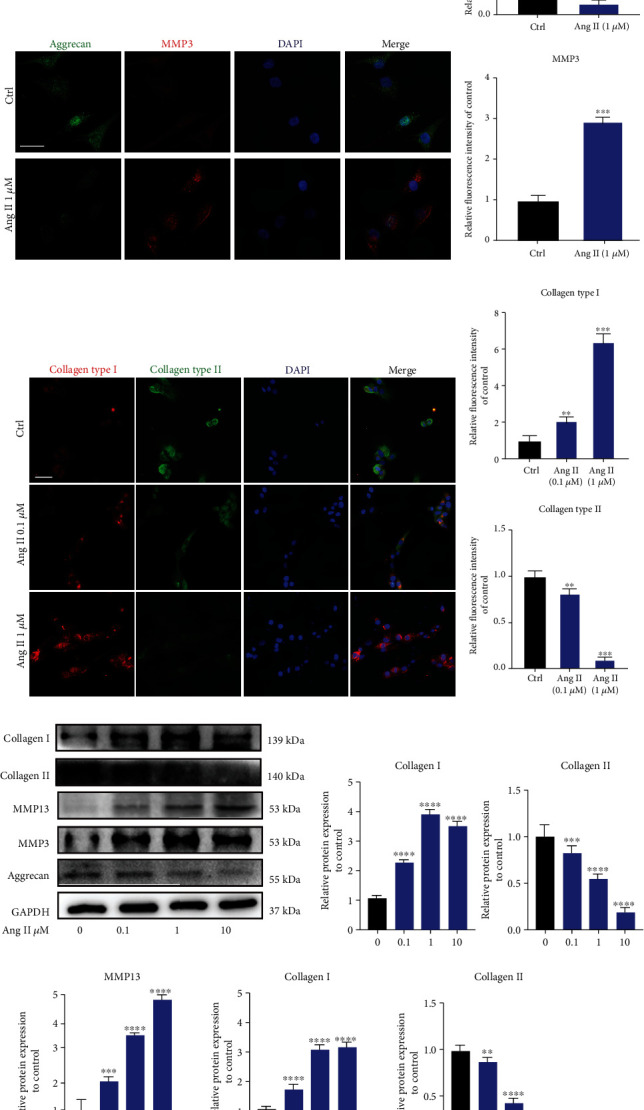
Angiotensin II induced degeneration and fibrosis phenotype in human NP cells in vitro. (a) Immunofluorescence results for the expression of aggrecan and MMP3 in human NP cells induced by Ang II. Scale bar = 50 *μ*m. (b) The relative expression of aggrecan and MMP3 was quantified (*n* = 3). (c) Immunofluorescence results for the expression of fibrosis-related proteins, collagen types I and II, in human NP cells induced by Ang II. Scale bar = 50 *μ*m. (d) Quantitative results for collagen types I and II were presented as graph (*n* = 3). (e, f) Western blot for the expression of IDD-related proteins in the human NP tissue (*n* = 3). *p* < 0.05, ^∗∗^*p* < 0.01, ^∗∗∗^*p* < 0.001, ^∗∗∗∗^*p* < 0.0001.

Decreased ECM (type II collagen and aggrecan) and elevated MMPs and type I collagen have been the typical features during IDD [7]. Therefore, in this present study, we evaluated the changes of these features. As shown by immunofluorescence analysis, human NP cells stimulated with Ang II expressed higher MMP 3 and lower aggrecan (Figures 5(a) and 5(b)). During the process of IDD, the gel-like NP tissue will possess fibrosis phenotype, with type II collagen being replaced by type I collagen [3, 7]. In addition, Ang II could promote tissue fibrosis [26, 27]. Thus, the expression of type I collagen was also evaluated, and the results suggested that Ang II significantly increased the expression of type I collagen (Figures 5(c) and 5(d)). Furthermore, consistent with the results of immunofluorescence analysis, western blot also confirmed that Ang II promoted degeneration and fibrosis phenotype in human NP cells in vitro (Figures 5(e) and 5(f)).

### 3.6. Angiotensin II Increased the ROS Level in Human NP Cells In Vitro

**Figure 6:**
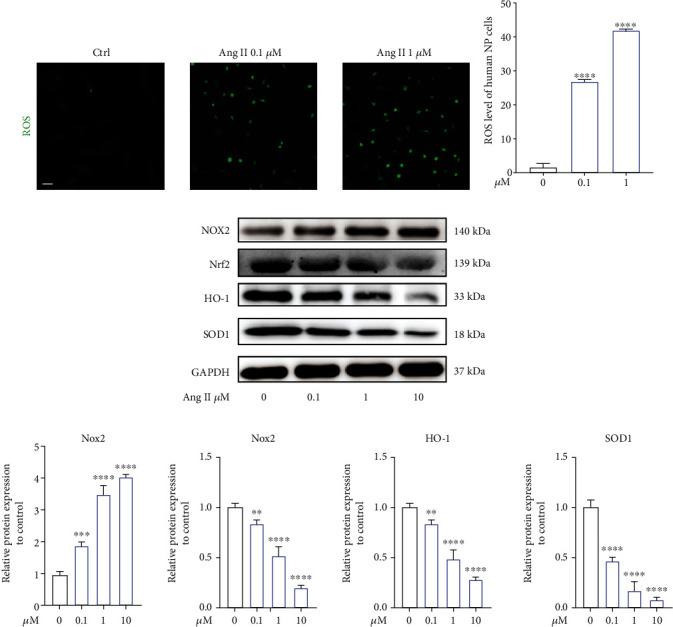
Angiotensin II increased the ROS level in human NP cells in vitro. (a) The level of intracellular ROS in human NP cells induced by Ang II. Scale bar = 50 *μ*m. (b) The relative amount of intracellular ROS was quantified (*n* = 3). (c) Oxidative stress-related proteins were presented by western blot (*n* = 3). (d) The results of western blot were quantified as graphs (*n* = 3). *p* < 0.05, ^∗∗^*p* < 0.01, ^∗∗∗^*p* < 0.001, ^∗∗∗∗^*p* < 0.0001.

ROS level frequently indicates intracellular oxidative conditions. Previous studies have revealed that Ang II could induce the release of reactive oxygen species (ROS) in cardiovascular diseases [28]. During IDD, excessive production of intracellular ROS will accelerate NP cell damage and inflammatory response [2]. As a result, we examined the effect of Ang II on human NP cells and found that Ang II dose-dependently enhanced the intracellular ROS level (Figures 6(a) and 6(b)). As molecular level, western blot demonstrated the decreased antioxidative stress-related proteins (Nrf2, HO-1, and SOD1) and increased prooxidative stress-related protein, NOX 2, induced by Ang II (Figures 6(c) and 6(d)).

### 3.7. Angiotensin II Promoted the Activation of NLRP3 Inflammasome in Human NP Cells In Vitro

**Figure 7:**
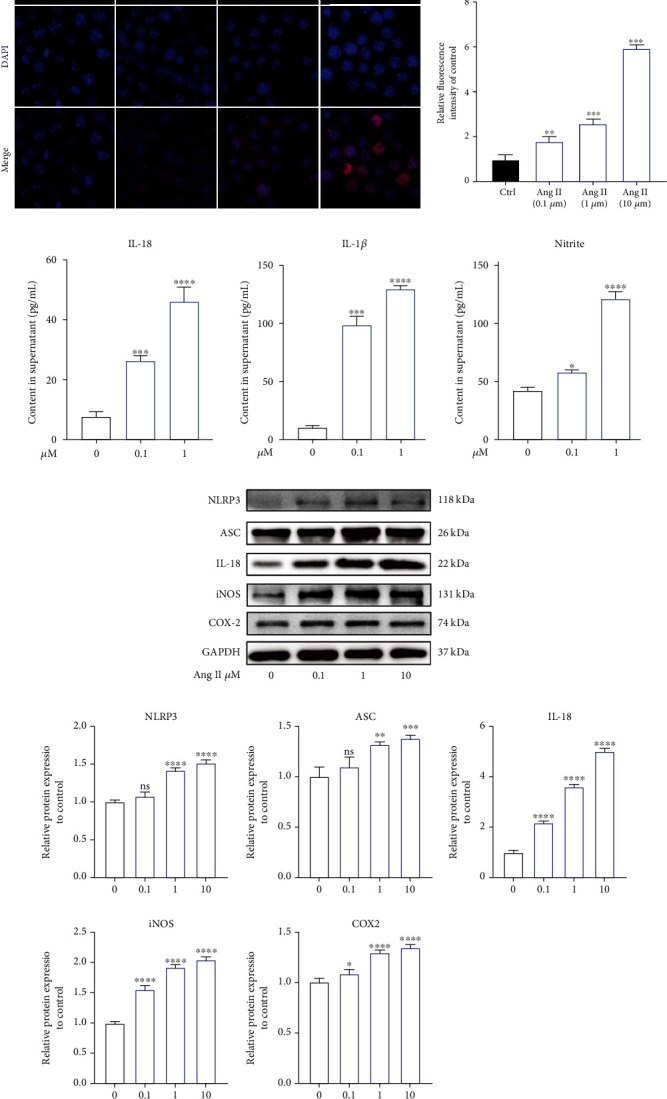
Angiotensin II promoted the activation of NLRP3 inflammasome in human NP cells in vitro. (a) Immunofluorescence results for the expression of NLRP3 in human NP cells induced by Ang II, *n* = 3. Scale bar = 50 *μ*m. (b) Ang II promoted the secretion of inflammation-related cytokines in human NP cells (*n* = 3). (c) The effects of Ang II on the expression of NLRP3 inflammasome-related proteins and inflammatory mediators in human NP cells (*n* = 3). (d) The results of western blot were quantified as graphs (*n* = 3). *p* < 0.05, ^∗∗^*p* < 0.01, ^∗∗∗^*p* < 0.001, ^∗∗∗∗^*p* < 0.0001.

The NLR pyrin domain-containing 3 (NLRP3) inflammasome is the crucial source of inflammation-related cytokines, such as IL-1*β* and IL-18 [29]. Notably, NLRP3 inflammasome has been indicated to involve in IDD recently [30, 31]. Therefore, we evaluated the activated effect of Ang II on NLRP3 inflammasome. As shown in the results, the ratio of NLRP3 inflammasome-positive NP cells was significantly increased by Ang II (Figure 7(a)). ELISA results revealed that Ang II also enhanced the secretion level of inflammatory cytokines, including Il-18, IL-1*β*, and NO (Figure 7(b)). The upregulated expressions of NLRP3 inflammasome-related proteins (ASC and IL-18) and inflammation-related proteins (iNOS and COX2) also confirmed the promotive effect of Ang II on the activation of NLRP3 inflammasome in human NP cells in vitro (Figures 7(c) and 7(d)). In addition, macrophage has also participated in IDD, with M1 macrophage increasing in the degenerated disc tissue [32]. We also evaluated the effect of Ang II on macrophage polarization and found that Ang II could increase the expression of M1 markers, iNOS, IL-1*β*, and TNF *α* and decrease the expression of M2 markers, CD 206, and YM1/2 (Supplementary Figure 2, A, B, and C). Collectively, we deduced that Ang II promoted the inflammatory reaction possibly via activating NLRP3 inflammasome and recruiting M1 macrophage in human NP cells.

### 3.8. Local Activated tRAS Existed in SHR Nucleus Pulposus Tissue In Vivo

**Figure 8:**
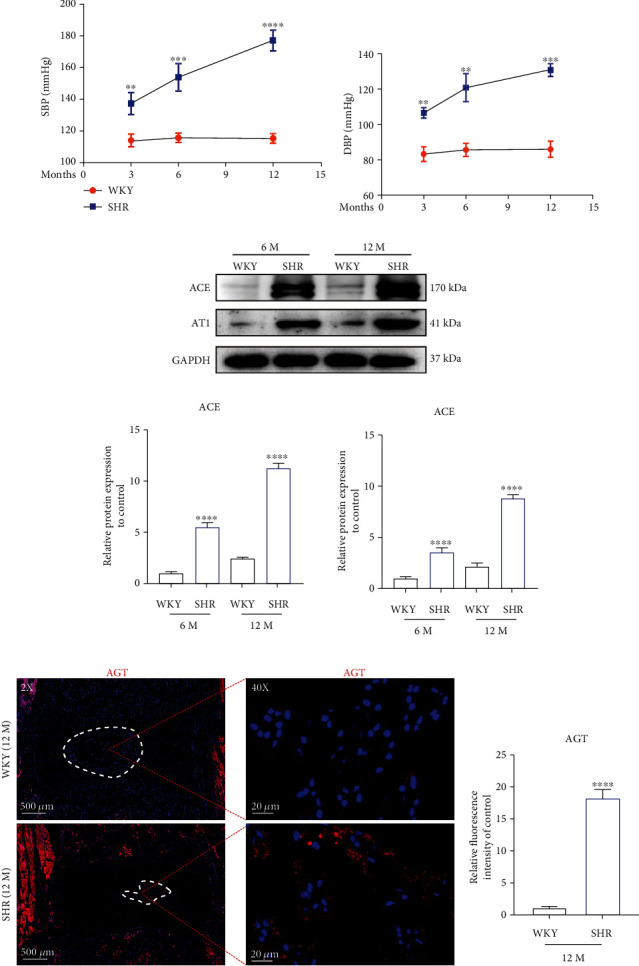
Systematic activation of RAS promoted the local expression of ACE in SHR nucleus pulposus tissue in vivo.(a, b) The blood pressure was measured with a noninvasive Tail-Cuff System Western (ALC-NIBP, Shanghai Alcott Biotech) at 3 M, 6 M, and 12 M, respectively, according to previous study [34]. (c) Western blot results for the expression of ACE and AT1 in the rat NP tissue at the age of 6 and 12 months, respectively (*n* = 3). (d) The results of western blot were quantified (*n* = 3). (e) Immunofluorescence results for the expression of AGT in the rat NP tissue at the age of 12 months (*n* = 3). (f) The results of immunofluorescence were quantified (*n* = 3). SBP: systolic blood pressure; DBP: diastolic blood pressure. *p* < 0.05, ^∗∗^*p* < 0.01, ^∗∗∗^*p* < 0.001, ^∗∗∗∗^*p* < 0.0001.

Spontaneously hypertensive rats (SHR) is characterized by systematic activation of RAS, which has been widely used in RAS-related research [33]. To further confirm the biological effects of tRAS on IDD, we introduced SHR, with Wistar-Kyoto (WKY) rats being the control group. Firstly, we evaluated the change of blood pressure of SHR and WKY rats and found relatively higher SBP and DBP in SHR than those in WKY rats since the age of 3 M (*p* < 0.05) (Figures 7(a) and 7(b)). Then, we explored the local expression of tRAS components in the disc tissue. As indicated by western blot, HR with age of 6 and 12 months showed higher expression of ACE and AT1 in the rat nucleus pulposus tissue compared to WKY rats (Figures 8(c) and 8(d)). Immunofluorescence analysis results also confirmed the higher local expression of AGT in SHR (12 months) NP cells than that in WKY rats, consistent with the results of proteomic analysis (Figures 8(e) and 8(f)). Collectively, systematic activation of RAS promoted the local activation of tRAS in SHR NP tissue in vivo. Interestingly, only the NP tissue was found positive cell that expressed ACE (Figure 7(a)). Collectively, SHR showed excessively activated tRAS in the intervertebral disc tissue, especially in the NP tissue.

### 3.9. Local Activated ACE/Ang II Accelerated Intervertebral Disc Degeneration in the Aging SHR Model

**Figure 9:**
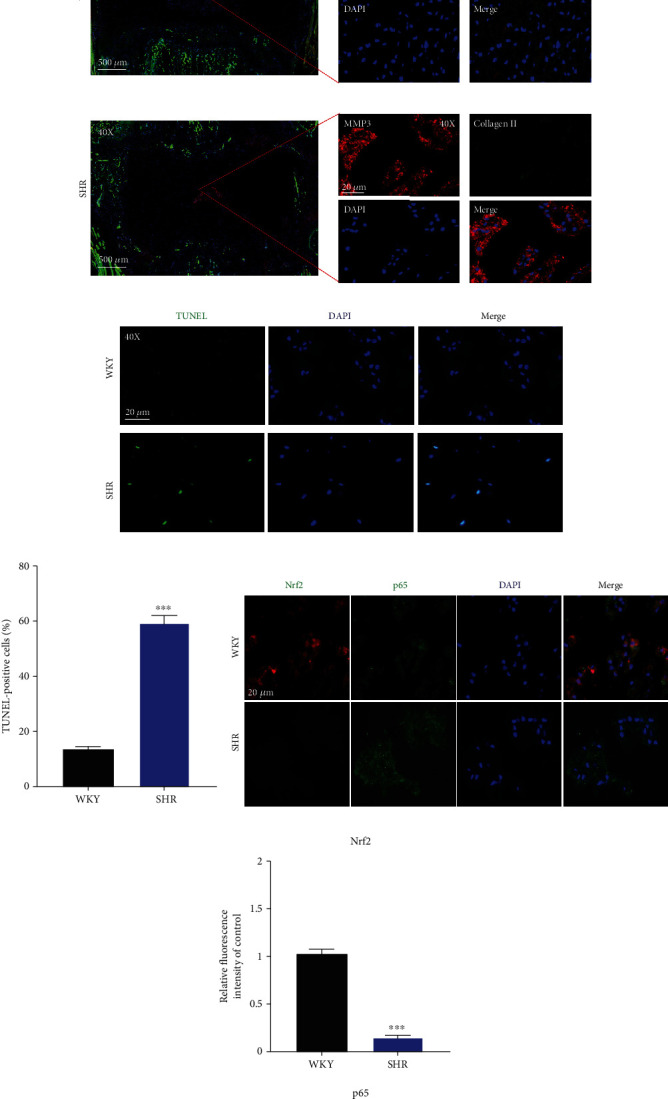
Local activated ACE/Ang II accelerated intervertebral disc degeneration in the aging SHR model. (a) SO-FG staining of intervertebral disc in the rat NP tissue at the age of 6 and 12 months, respectively (*n* = 3). (b) Immunofluorescence results for the expression of MMP3 and collagen II in the rat NP tissue at the age of 12 months (*n* = 3). (c, d) TUNEL staining and quantified results for apoptotic NP cells in the rat NP tissue at the age of 12 months, respectively (*n* = 3). (e, f) Immunofluorescence results for the expression of Nrf2 and p65 in the rat NP tissue at the age of 12 months (*n* = 3). *p* < 0.05, ^∗∗^*p* < 0.01, ^∗∗∗^*p* < 0.001, ^∗∗∗∗^*p* < 0.0001.

SHR and WKY rats with the age of 6 months and 12 months were used to evaluate the effect of local activated tRAS on IDD. As shown in Figure 9, SO-FG staining revealed that the NP tissue of SHR showed decreased proteoglycan content at both the age of 6 and 12 months (Figure 9(a)). Notably, at 12 months, the NP-AF boundary became not clear in SHR, with less NP tissue, compared to that in WKY rats (Figure 9(a)). Immunofluorescence analysis revealed that compared to WKY rats, there was higher expression of MMP 3 and lower expression of collagen type II in SHR (Figure 9(b)). The TUNEL assay of THE disc tissue also suggested higher ratio of positive cells (Figures 9(c) and 9(d), *p* < 0.05). Nuclear tor erythroid 2-related factor-2 (Nrf2)/NF-*κ*B signal cascade has been the critical pathway in IDD [2]. Therefore, we further examined the expression of these two proteins and found that at 12 months, SHR expressed higher protein level of p65 and lower protein level of Nrf2, compared to those in WKY rats (Figures 9(e) and 9(f), *p* < 0.05). Taken together, local activated ACE/Ang II could accelerate IDD in aging SHR, and Nrf2/NF-*κ*B signal cascade could be involved.

## 4. Discussion

IDD is an chronic inflammation process, with plentiful activated inflammation-related cytokines (COX-2, iNOS, IL-1*β*, TNF-*α*) and ECM degradation-related enzymes (MMP3/13, ADAMTS4/5/7) being excessively expressed [35]. Thus, strategies targeting at attenuating the inflammation response in IVD may prevent or postpone the onset of IDD. In the past few decades, increasing evidence has suggested that RAS shows its biological effects not just limited in the cardiovascular system. Various local tissue RAS (tRAS) have been identified in multiple tissues, including the brain, spinal cord, skin, kidney, heart, and bone, participating in inflammation, senescence, apoptosis, and fibrogenesis [16, 26, 36, 37]. In recent years, tRAS has also been established in both bovine and human intervertebral disc tissue, especially in the degenerated disc tissue [17, 18]. However, the exact of tRAS and IDD remains unknown.

In this present study, proteomic analysis, molecular biology analysis, and animal model were used, and the results revealed that (1) tRAS was activated in the human degenerated intervertebral disc tissue. (2) Ang II could decrease the cell viability of human NP cells, promote NP cell apoptosis, senescence, oxidative stress, and NLRP3 activation, and recruit M1 macrophage in human NP cells. (3) Ang II could trigger degeneration and fibrosis phenotype in human NP cells. (4) Local activated ACE/Ang II could accelerate IDD in aging SHR.

Cell senescence and apoptosis are two kinds of normal biological response to exogenous or endogenous stress and damage [38]. Human intervertebral disc tissue is the largest avascular tissue in the body, with high susceptibility to ischemia, hypoxia, and nutrient deprivation, and all these factors will trigger NP cell senescence and apoptosis [39]. NP cells have been the major cells that maintain the normal function of disc. Therefore, NP tissue and cells were used in this present study. We firstly evaluated the correlation between Ang II and IDD using the human intervertebral disc tissue. The results found increased apoptotic rate of NP cells and expression of MMP13, as well as increased expression of tRAS components, such as ACE and AT1. The consistent changes above suggested a potentially close correlation between tRAS and IDD. In vitro experiment also confirmed the positive correlation between activated tRAS and IDD. SA-*β*-gal and p53 are the two typical senescence-related markers [40]. We found that Ang II increased the expression of SA-*β*-gal and p53 in human NP cells, as well as increased the amount of *γ*H2AX distribution over nuclear DNA. Klotho is a newly discovered antiaging gene, and deficiency in Klotho can result in senescence-like phenotypes [41]. Previous study also suggested an antagonistic relationship between Klotho and Ang II [37]. Therefore, we examined the expression of Klotho and found decreased expression of Klotho in Ang II-induced human NP cells. In addition, Ang II also caused increased ratio of apoptotic cells and decreased MMP in human NP cells. Taken together, local activated tRAS in the human disc tissue could trigger senescence and apoptosis to accelerate IDD.

Another major characteristic of IDD is excessive accumulation of MMPs and decreasing expression of aggrecan and type II collagen. In general, aggrecan and type II collagen have been the primary components in the intervertebral disc tissue, which are responsible for maintaining its high-water content feature. However, the abnormal expression of MMP3/13 will result in an imbalance between anabolism and catabolism of ECM and eventually the loss of intervertebral height [42]. In this present study, we also examined the effects of Ang II on MMPs and ECM. As shown in the results, Ang II increased the expression of MMP3/13 and decreased the expression of aggrecan and collagen type II. In addition, during IDD, the type II collagen will be gradually replaced by type I collagen [3, 7]. In fact, increasing studies have reported that Ang II induces tissue fibrosis [27, 43]. We also found that Ang II caused the increased expression of type I collagen in NP cells. The results above suggested that Ang II could induce degeneration and fibrosis phenotype in human NP cells in vitro.

Oxidative stress and inflammation reaction have been the major causes to IDD. Excessive production of inflammatory cytokines and ROS will trigger or aggravate cell senescence, apoptosis, and ECM degradation to accelerate IDD [30]. In fact, the two biological progresses frequently cooccurred. Proinflammatory cytokines, such as IL-1*β*, could enhance the level of intracellular ROS [44, 45]. Likely, high-level ROS also could increase the release of inflammatory factors [46]. Firstly, we found that Ang II dose-dependently enhanced intracellular ROS, as well as decreased the expression of Nrf2, HO-1, and SOD1. NLRP3, as one of the most studied inflammasomes, has been recently reported to participate in the inflammatory responses during IDD [47, 48]. In this present study, we found that Ang II activated the NLRP3 pathway, followed by increased production of proinflammatory cytokines. In addition, the expression of inflammatory mediators, COX-2 and iNOS, were also enhanced. In addition, we also found that Ang II could induce macrophage M1 polarization. Previous studies have suggested that M1 macrophages could promote degenerative phenotypes in rat NP cells [49]. Furthermore, the mitochondrial pathway could implicate in the activation of NLRP3, and the generation of ROS and NLRP3 activation would be suppressed when mitochondrial activity was inhibited [50]. Therefore, combined the results above that Ang II could decrease MMP of mitochondrion, we deduced that Ang II could induce macrophage infiltration, inflammatory response, and oxidative stress and lead to mitochondrial dysfunction and NP cell injury. Damaged mitochondrion would further generate ROS. Subsequently, overproduction of ROS would trigger the activation of NLRP3 and the release of inflammatory cytokines. Thus, a vicious feedback among Ang II, mitochondrion, and NLRP3 will be established, accelerating NP cell injury. Agents targeting at these three points may facilitate to further treatment of IDD.

To further confirm the biological effects of tRAS on IDD, we introduced spontaneously hypertensive rats (SHR) which is characterized by systematic activation of RAS, with WKY rats as the control group [33, 34]. Firstly, we provided the evidence that local overactivated tRAS existed in the SHR NP tissue, but not in the AF tissue. Then, we found that aging SHR showed decreased proteoglycan content and reduced NP tissue compared to WKY rats. In addition, compared to WKY rats, there were higher ratio of apoptotic cells, higher expression of MMP3, and lower expression of collagen I in the SHR NP tissue. The results were consistent with the results of in vitro experiments above, which suggested that SHR had higher susceptibility to IDD. Therefore, a potential correlation between hypertension and IDD may exist, which is consistent with previous clinical studies that hypertension was a risk factor to low back pain [9, 51]. In addition, treatment with RAS inhibitors has been shown anti-inflammatory effects in arthritis, a similar disease to IDD [52]. Therefore, tRAS may be a novel therapeutic target for IDD. In addition, we found that SHR at 12 months expressed higher protein level of p65 and lower protein level of Nrf2, which have been reported to correlate with oxidative stress and inflammation during IDD [2]. Taken together, local activated ACE/Ang II could accelerate IDD in aging SHR, and Nrf2/NF-*κ*B signal cascade could be involved. However, the further exact mechanism between tRAS and IDD remains to be identified.

Several limitations must be acknowledged. Firstly, the clinical sample size in this present study was still limited. However, to minimize the potential influence factors, we confirmed the correlation between tRAS and IDD from perspectives of proteomic analysis, western blot, and immunohistochemistry analysis. In addition, human NP cells induced by IL-1*β* and TNF*α* also showed consistent results. Therefore, we could conclude that the activated tRAS exists in the human degenerated intervertebral disc tissue. Secondly, SHR has been reported to show overactivated systematic RAS. Although we have also confirmed the existence of activated tRAS in the SHR NP tissue, it will be better to examine the relationship between Ang II and IDD using animal models that specifically highly expressed Ang II gene. Thirdly, therapeutic experiments against the ACE/Ang II axis were not shown in this present study. In fact, we are now performing related experiments, and further outcomes will be presented in this journal if possible.

## 5. Conclusion

Degenerated intervertebral disc tissue showed excessively activated tRAS. Local activation of tRAS could induce NP cell senescence, apoptosis, oxidative stress, and inflammatory reaction to cause degeneration and fibrosis phenotype in human NP cells. These biological effects of Ang II on NP cells may be achieved via Nrf2/p65/NLRP3 pathway. The results will provide new information for future investigation of hypertension and IDD.

## Figures and Tables

**Table 1:** Sequences of the primers for RT-qPCR.

Gene	Primer	Sequence
ACE	Forward	CTTTGACGGAAGCATCACC
	Reverse	GGCACATTCGCAGGAAC

AT1	Forward	GCTGTCATCCACCGAAA
	Reverse	GGAACAAGAAGCCCAGAA

GAPDH	Forward	TGACCACAGTCCATGCCATC
	Reverse	GACGGACACATTGGGGGTAG

## Data Availability

Data are available when required.

## Supplementary Materials

Supplementary Materials Supplementary Figure 1: illustration of the study workflow LC–MS/MS: liquidchromatography–tandem mass spectrometry. Supplementary Figure 2: Ang II promoted M1 polarization of RAW264.7. (a, b) Ang II increased the expression of M1 marker, iNOS, and decrease the expression of M2 marker, CD 206. (c) RT-qPCR revealed that Ang II increased the expression of M1 markers, IL-1*β* and TNF *α*, and decrease the expression of M2 markers, CD 206 and YM1/2. Scale bar = 20 *μ*m.
