# Supplementary material for: Tissue Renin-Angiotensin System (tRAS) Induce Intervertebral Disc Degeneration by Activating Oxidative Stress and Inflammatory Reaction
Source: Oxid Med Cell Longev. 2021 Aug 6;2021:3225439. doi: 10.1155/2021/3225439 (PMC8369181; doi:10.1155/2021/3225439)
Supplement: Supplementary Materials — Supplementary Figure 1: illustration of the study workflow LC–MS/MS: liquidchromatography–tandem mass spectrometry. Supplementary Figure 2: Ang II promoted M1 polarization of RAW264.7. (a, b) Ang II increased the expression of M1 marker, iNOS, and decrease the expression of M2 marker, CD 206. (c) RT-qPCR revealed that Ang II increased the expression of M1 markers, IL-1β and TNF α, and decrease the expression of M2 markers, CD 206 and YM1/2. Scale bar = 20 μm. [file 3225439.f1.docx]

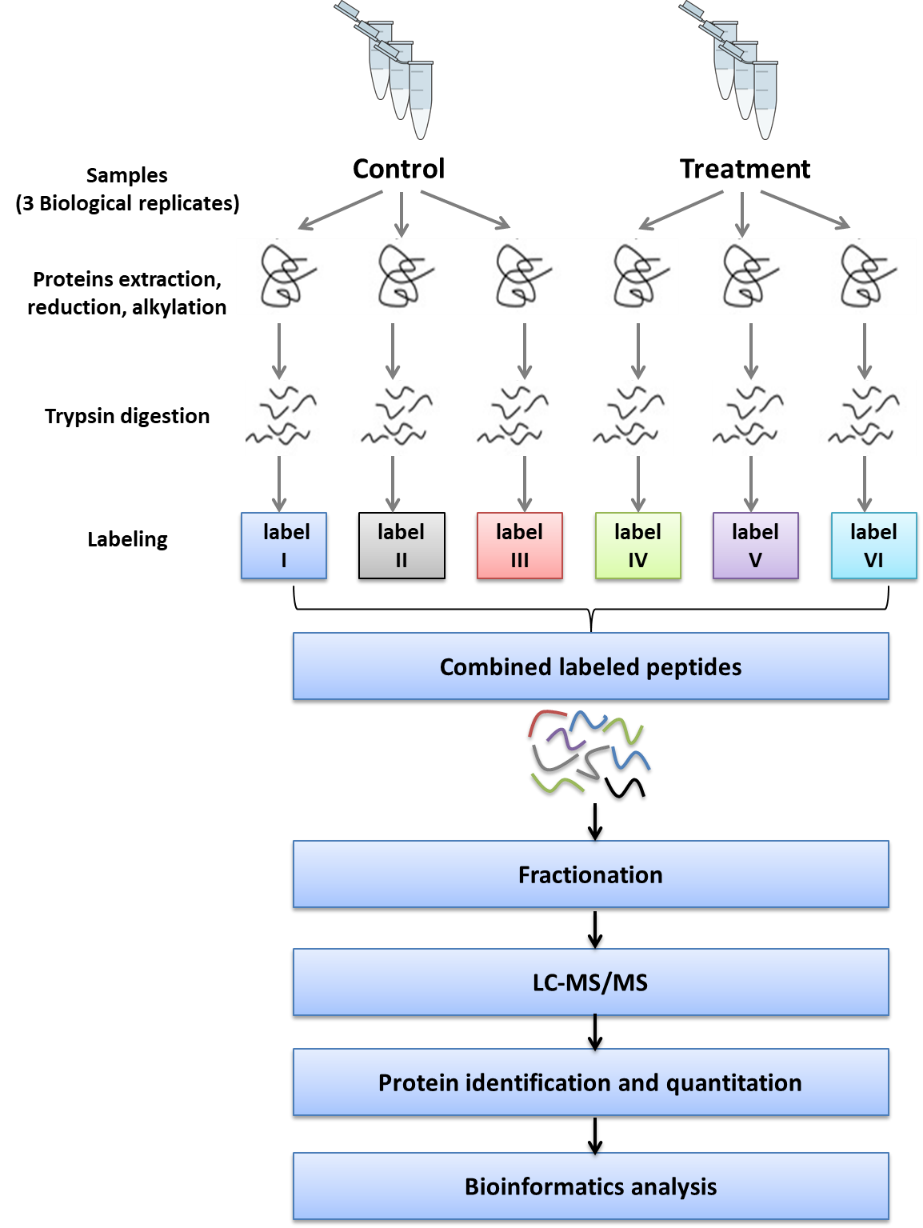


Supplementary Figure 1 Illustration of the study workflow

LC–MS/MS: liquidchromatography–tandem mass spectrometry


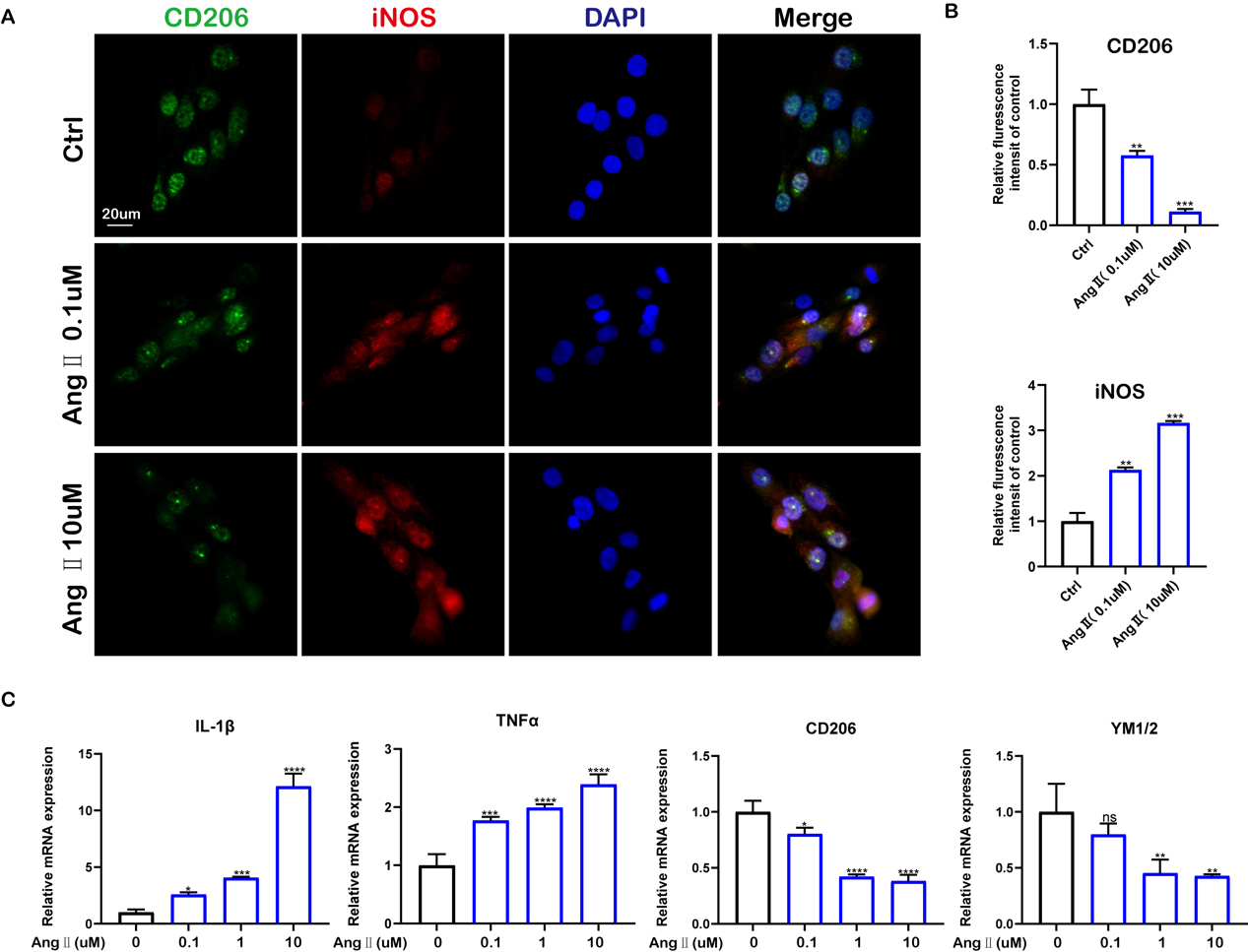


**Supplementary Figure 2 Ang II promoted M1 polarization of RAW264.7** A and B: Ang II increased the expression of M1 marker, iNOS, and decrease the expression of M2 marker, CD 206. C: RT-qPCR revealed that Ang II increased the expression of M1 markers, IL-1β and TNF α, and decrease the expression of M2 markers, CD 206 and YM1/2. Scar bar=20um.
